# Supplementary material for: The causal role of circulating inflammatory markers in osteoporosis: a bidirectional Mendelian randomized study
Source: Front Immunol. 2024 Jul 18;15:1412298. doi: 10.3389/fimmu.2024.1412298 (PMC11291241; doi:10.3389/fimmu.2024.1412298)
Supplement: Supplementary file 1 [file DataSheet_1.zip › supplementary material/CIM-OSTEOPOROSIS /GCST90274814.csv_forest.pdf]

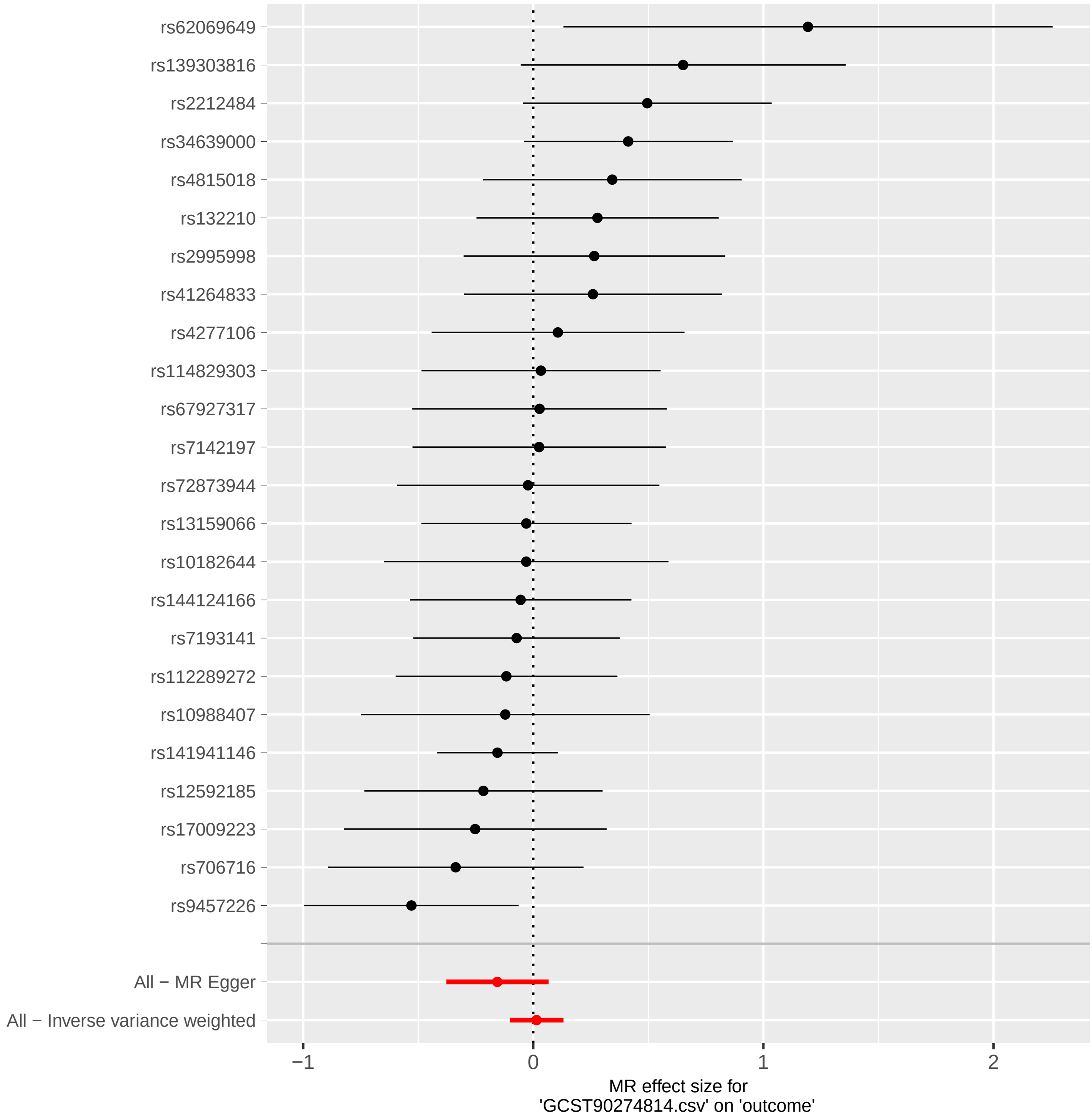

rs62069649
rs139303816
rs2212484
rs34639000
rs4815018
rs132210
rs2995998
rs41264833
rs4277106
rs114829303
rs67927317
rs7142197
rs72873944
rs13159066
rs10182644
rs144124166
rs7193141
rs112289272
rs10988407
rs141941146
rs12592185
rs17009223
rs706716
rs9457226
All − MR Egger
All − Inverse variance weighted
−1
0
1
2
MR effect size for
'GCST90274814.csv' on 'outcome'
